# Is this patient really “(un)stable”? How to describe cardiovascular dynamics in critically ill patients

**DOI:** 10.1186/s13054-019-2551-1

**Published:** 2019-08-06

**Authors:** Jean-Louis Vincent, Maurizio Cecconi, Bernd Saugel

**Affiliations:** 1 Department of Intensive Care, Erasme Hospital, Université libre de Bruxelles, 1170 Brussels, Belgium; 2 Department of Anesthesia and Intensive Care Units, Humanitas Research Hospital, 20089 Milan, Italy; 3 Department of Anesthesiology, Center of Anesthesiology and Intensive Care Medicine, University Medical Center Hamburg-Eppendorf, 20246 Hamburg, Germany

Earlier this week during rounds in the intensive care unit, a resident reported, “Mr S. became hemodynamically unstable so we had to give norepinephrine.” Later, another resident described a patient with acute respiratory distress syndrome who had been on renal replacement therapy for the last 36 h as being, “hemodynamically stable under 1 μg/kg/min of norepinephrine.” This led us to reflect on the meanings of these two words—“stable” and “unstable”—when describing cardiovascular dynamics in critically ill patients.

The terms hemodynamically “stable” and “unstable” are used frequently but what do they actually mean? And are they appropriate or even correct? Can a critically ill patient ever really be accurately described as being stable or unstable? A stable condition can be defined as a situation that does not change substantially over time. But surely all critically ill patients are per se *un*stable as, by the very nature of being critically ill, their physiological variables—including cardiovascular dynamics—change frequently over time [1]. Although vital signs can appear stable when a patient is receiving organ support, the patient is still critically ill. Terminology in such patients needs to be precise, and vague descriptive terms should be avoided. Indeed, there are no generally accepted and uniform definitions of the conditions stable and unstable, and the same patient may be classified as stable or unstable by different doctors and nurses depending on their clinical judgment, experience, and knowledge of the patient’s clinical course. In Fig. 1, we propose some clinical scenarios that demonstrate these problems with the use of the words “stable” and “unstable” when describing cardiovascular dynamics in critically ill patients.

**Fig. 1:**
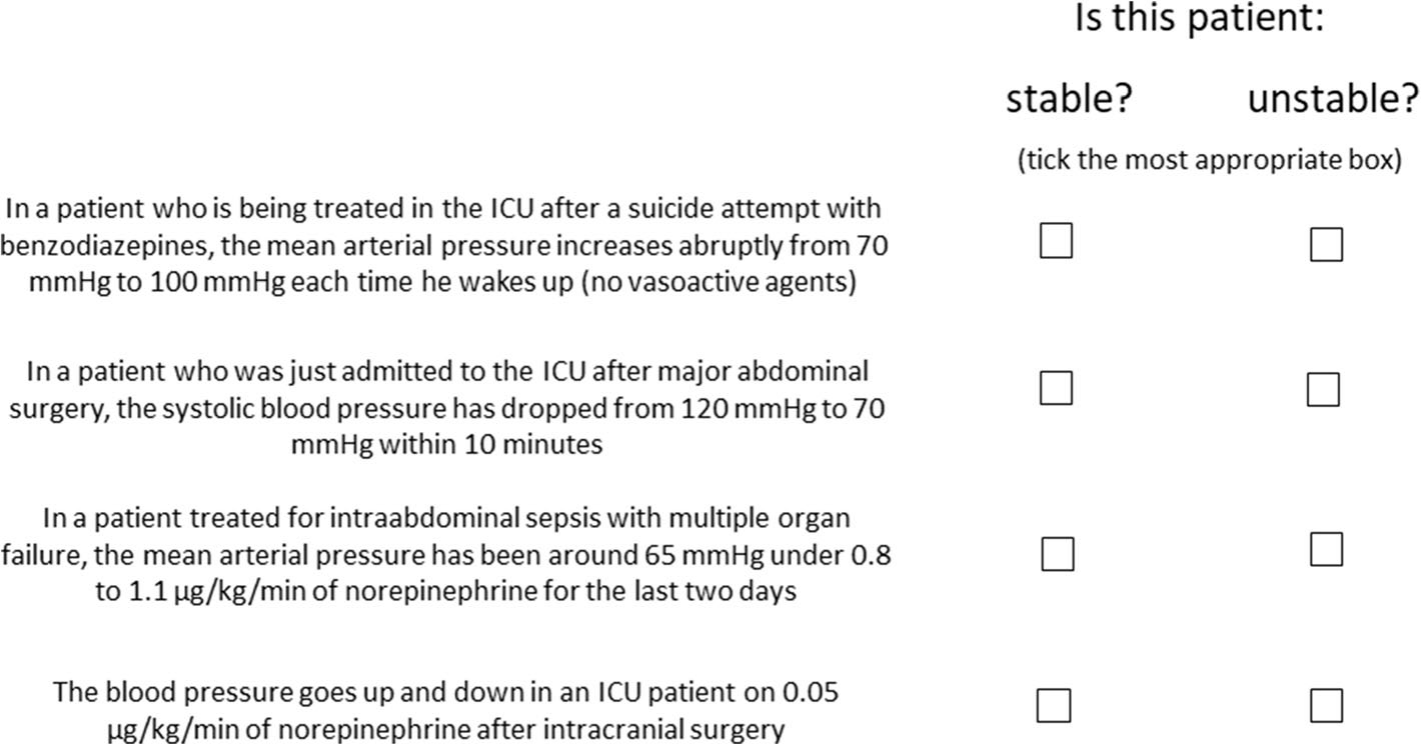
What is hemodynamic (in)stability

So, how should we describe these patients? To the first resident, we suggested that Mr. S. who had become “hemodynamically unstable” had actually developed circulatory shock and that this was the preferred term. To further describe the cardiovascular dynamics in this and similar patients, available objective criteria (blood pressure, cardiac output, rate of vasopressor or inotrope) should be used.

For the second resident, the problem is perhaps more related to interpretation than definition. The word “stable” often has positive connotations when used to refer to patient condition. When the resident described his patient as “being stable,” everyone agreed, knowing that this meant there had been no acute change in the patient’s condition or treatment. In fact, the patient still had profound circulatory shock. Using the word “stable” in such patients may even create a false sense of security for the care team, such that they begin to accept the critical state of the patient as “normal” and lessen efforts to try and resolve the serious condition. Additionally in this case, when the relatives called anxiously to get some news and the nurse said “the situation is stable,” this gave them the impression that their loved one was not getting worse, thus offering them some hope of recovery. Without further more detailed explanation, relatives may not understand that being stable in such critical conditions actually means the patient is not getting better and his/her chances of a positive outcome are likely getting worse. Indeed, the duration of shock is an important prognostic factor [2, 3].

Although widely used among physicians and frequently present in the literature, the words “stable” and “unstable” to describe cardiovascular dynamics in critically ill patients can have different meanings to different people and in different situations, making them confusing word choices that should be avoided. We must be careful and precise with our choice of words to colleagues, patients, and families and avoid vague terms that could be misinterpreted. The word “stable” should not be used to describe a condition that remains critical, and “hemodynamic instability” should be described using objective criteria such as blood pressure, cardiac output, or vasopressor dose.

## Data Availability

Not applicable.
